# Carry-Over Effects of Desiccation Stress on the Oxidative Status of Fasting Anuran Juveniles

**DOI:** 10.3389/fphys.2021.783288

**Published:** 2021-12-02

**Authors:** Marko D. Prokić, Tamara G. Petrović, Branka R. Gavrilović, Svetlana G. Despotović, Jelena P. Gavrić, Ana Kijanović, Nataša Tomašević Kolarov, Tanja Vukov, Tijana B. Radovanović

**Affiliations:** ^1^Department of Physiology, Institute for Biological Research “Siniša Stanković”, National Institute of the Republic of Serbia, University of Belgrade, Belgrade, Serbia; ^2^Department of Evolutionary Biology, Institute for Biological Research “Siniša Stanković”, National Institute of the Republic of Serbia, University of Belgrade, Belgrade, Serbia

**Keywords:** amphibians, antioxidant system, *Bombina variegate*, development, food deprivation, oxidative stress, pond drying, yellow belly toad

## Abstract

Amphibians are sensitive to deteriorating environmental conditions, especially during transition to a terrestrial environment which is full of uncertainties. Harsh conditions, such as desiccation during earlier stages, affect different larval traits with possible carry-over effects on juvenile and adult life histories. The first consequences of the effects can be seen in juveniles in the challenges to find food and the ability to survive without it in a terrestrial habitat. Body size and the internal energy reserves acquired during the larval phase play an important role in this period. Herein, we tested how different water regimes (low water availability, desiccation and constant high-water availability) during larval development reflect on the oxidative status and ability of yellow belly toad (*Bombina variegata*) juveniles to endure short-term fasting. The desiccation regime significantly reduced the body size of metamorphs. The same was observed after 2 weeks of fasting, while the feeding treatment reduced differences mostly in the body mass of individuals from different water regimes. This was the result of a greater gain in mass in juveniles pre-exposed to desiccation. Pre-exposure to desiccation also modified the parameters of the antioxidant system (AOS) under feeding conditions, leading to higher values of superoxide dismutase, glutathione reductase and glutathione S-transferase, glutathione and sulfhydryl group concentrations, and lower glutathione peroxidase in comparison to juveniles reared under constant water. The increase in the AOS of juveniles can be considered as a physiological carry-over effect of desiccation, probably as the result of compensatory growth and/or earlier exposure to chronic stress. However, water levels during larval development did not exert significant effects on the oxidative status of juveniles subjected to food unavailability. Fasting juveniles, both control and desiccated, were exposed to oxidative stress, significantly higher lipid peroxide concentrations, lower superoxide dismutase, glutathione peroxidase, glutathione S-transferase, glutathione and sulfhydryl group values in comparison to feeding individuals. The lack of food in juvenile anurans activated the AOS response in the same manner, regardless of body size and stress pre-exposure, suggesting that the generally accepted hypothesis about the influence of metamorphic body size on the fitness of the postmetamorphic stage should be tested further.

## Introduction

Shifts in environmental conditions caused by different climate events can have profound effects on an organism’s phenotype, fitness, reproductive success and life span. To manage these changes, the organism needs to optimize the trade-offs in time and resource allocation (Metcalfe and Alonso-Alvarez, 2010). Amphibian larvae are often challenged by fluctuations in the environment (Gavrilović et al., 2021; Gavrić et al., 2021). Increased global temperatures and anthropogenic habitat destruction augment the pond desiccation process, which alters larval development (Burraco et al., 2020a). A low water volume limits larval movement, food sources, increases density and predator exposure (Kohli et al., 2019). Under such unfavorable conditions, larvae activate mechanisms of phenotypic plasticity response [the hypothalamic−pituitary−interrenal (HPI) axis and increased thyroid hormone levels] and accelerate the developmental process that allows them to leave the aquatic environment (Burraco et al., 2017; Ruthsatz et al., 2020). Phenotypic-developmental plasticity is particularly critical for amphibians and other species with complex life cycles as it allows the organism to react to specific environmental inputs (Simon et al., 2015; Agudelo-Cantero and Navas, 2019). The shortening of hydroperiods was shown to be an environmental factor that can result in adaptive phenotypic plasticity in amphibian larvae development (Newman, 1989; Denver, 1997; Richter-Boix et al., 2011). Even though at the moment plastic response is beneficial and allows for a niche shift, it affects the timing of metamorphosis, upsets the sensitive energy balance (between growth, development, storage and maintenance) and changes the body-size threshold, which is crucial for the survival of postmetamorphic individuals. The potential carry-over effects on postmetamorphs due to the allocation of energy sources toward premature metamorphosis can limit the larval energy required for growth, the antioxidant and immune systems (Gervasi and Foufopoulos, 2007; Prokić et al., 2019; Sinsch et al., 2020). The most studied trade-off between the aquatic and terrestrial life stages is between body size and developmental time (Newman, 1989; Denver, 1997). Accelerated development is associated with a smaller size at metamorphosis (Nicieza et al., 2006; Lind and Johansson, 2011; Székely et al., 2017, 2020). The body size and energy reserves of metamorphosed individuals entering a novel habitat are generally considered to be positively correlated with fitness, survival of the first year of life, hibernation and size at maturity (Álvarez and Nicieza, 2002; Sinsch et al., 2020). These traits are especially crucial when juveniles face the uncertainty of finding food due to a lack of foraging experience and adaptation to new food sources (Richter-Boix et al., 2006). If the environmental conditions are favorable, smaller juveniles could try to achieve optimal body size through compensatory growth. But if they are faced with the energy limitations imposed by food deprivation, juveniles need to switch to the mode of using internal energy stores, to change energy metabolism and to downsize all processes that are non-essential for survival (Moreira et al., 2018; Prokić et al., 2021). Operating a biological system under a food-deprived state results in physiological modifications in mitochondria activity and increased levels of oxidative stress (Salin et al., 2018; Metcalfe and Olsson, 2021). The state of oxidative stress is associated with damage of macromolecules through lipid peroxidation, protein denaturation, nucleic acid damage and impaired cell function, with consequences on subsequent life events (Halliwell and Gutteridge, 2015). Therefore, the response of an antioxidant system (AOS) is a pivotal process in preventing oxidative stress damage (Metcalfe and Alonso-Alvarez, 2010). The price of operating and maintaining this system is related to the greater use of nutritional reserves (Eikenaar et al., 2018; Janssens and Stoks, 2018; Prokić et al., 2018). With this in mind and according to the life history theory, juveniles with a smaller body should experience a greater trade-off in oxidative stress under conditions of limited energy such as fasting. This is to be expected because of the lower capability of smaller individuals to invest in antioxidant protection in comparison to larger organisms. However, as the abrupt ontogenetic switch points caused by pond drying conditions can additionally affect a set of modifications in oxidative metabolism necessary for the metamorphosis (Allen, 1991; Guxens et al., 2012; Moreira et al., 2021) this may reflect on juveniles AOS in response to fasting conditions. Adaptive reaction to stressful conditions can in some cases enhance cellular response, leading to a hormetic response (Oliveira et al., 2018).

The aims of this study were to investigate (i) the effects of exposure to an environmental stressor (desiccation) during anuran larval development on body size and the oxidative status of metamorphosed individuals, and (ii) the possible trade-off in body size (a smaller body size) induced by desiccation reflected on fitness—on the oxidative stress of juvenile individuals under the energy condition imposed by fasting. To test this, we manipulated the water levels, imitating pond-drying conditions that affected the growth of yellow belly toad (*Bombina variegata*) larvae. *B. variegata* was chosen as a model organism for examining the carry-over effects because of the considerable variations in body size of juveniles in natural populations (Biancardi and Di Cerbo, 2010; Schäfer et al., 2018) and the ability to reproduce and develop in ponds with an increased risk of desiccation before metamorphosis (Hartel et al., 2007). Starting from the results of our previous studies and the above mentioned, we assumed that (i) pre-exposure to desiccation would result in a lower body size and modification of the oxidative status of juvenile toads, and that (ii) food deprivation would induce oxidative stress in juvenile individuals, resulting in greater oxidative damage in individuals undergoing developmental desiccation stress.

## Materials and Methods

### Experimental Setup

The eggs of five clutches of *Bombina variegata* (yellow-bellied toad) were obtained in May 2020 from different ephemeral ponds located in the forests of the National Park Fruška Gora in Serbia. After transport to the laboratory, the eggs were reared in separate containers filled with 2 L of dechlorinated tap water until developmental stage 25 (Gosner, 1960). At this stage, 30 larvae from each clutch were randomly separated into six plastic containers (42 × 32 × 21.5 cm) filled with 10 L of water to a depth of 9.4 cm. Five individuals per container were chosen so as to avoid any potential density effects.

### Desiccation Experiment

The desiccation experiment was initiated at mid-prometamorphosis when the larvae reached Gosner stage 34. During this stage, larvae display maximum capacity for enhanced development (Gomez-Mestre et al., 2013). Individuals were placed in two water regimes: a constant high-water regime, which served as the control (with a 10-L volume and a depth of 9.4 cm, housing 30 tadpoles), and the desiccation water regime (with a volume of 5 L and a depth of 4.7 cm, housing 30 tadpoles). The water from the containers was renewed every 4 days, while the larvae were fed *ad libitum* every 2nd day with one tablet of commercial fish food (Tetra TabiMin^®^, Tetra GmbH, Melle, Germany) per container. The progress of larval development was checked daily. From stage 42 (Gosner, 1960), a wet terrestrial substrate was added. When all larvae completed metamorphosis, they were photographed with a Sony DSC-F828 digital camera at 3,264 × 2,448 pixel resolution, for calculation of the snout-to-vent length (SVL) using tpsDig2 software. Body mass (BM) was measured with a Kern EW 220-3NM precision balance (Kern & Sohn Balinge Germany, with an error of measurement of 0.001 g). Log-transformed data for BM and SVL was used to obtain the body condition index (BCI) (Labocha et al., 2014).

### Fasting Experiment

At the juvenile stage, random individuals from the control and desiccation groups were divided into 2-week-long fasting and/or feeding treatments (15 individuals per group). Four different treatment-groups were formed as follows: juveniles that developed in constant high water under normal *ad libitum* feeding (CC—control control); juveniles that developed in constant high water and were exposed to fasting (CF—control fasting); juveniles that developed under desiccation and normal *ad libitum* feeding (DC—desiccation control); juveniles that developed under desiccation and were exposed to fasting (DF—desiccation fasting). Juveniles were kept separately in 3-L plastic boxes (one individual per box), to avoid intraspecific competition and to follow the biometric parameters of each individual. Tubifex was provided as a food source for the animals in the feeding groups. Boxes were cleaned of feces twice a day to prevent possible coprophagia. The wet terrestrial habitat and water in Petri dishes were changed twice a week.

Individuals in the fasting group were maintained on the fasting regimen for 14 days, which is a realistic period of short-term food deprivation that might be experienced by newts in nature (Geise and Linsenmair, 1988; Scott et al., 2007). Laboratory conditions were maintained with a natural photoperiod (14 h light:10 h dark) and a room temperature of 20°C. All individuals survived the fasting/feeding experiment. At the end of the experiment, the juveniles were sacrificed by immersion in liquid nitrogen and kept at –80°C until further analysis (Underwood et al., 2013).

### Sample Processing and Biochemical Measurements

The whole bodies of juveniles used for analyses of oxidative stress parameters were minced to obtain as many homogeneous samples as possible. Part of the samples was taken for determination of thiobarbituric acid-reactive substance (TBARS). According to the assay of Rehncrona et al. (1980), about 0.2 g of the sample was homogenized and sonicated at pH 7.4 in 10 volumes of ice-cold Tris-HCl buffer. The sonicates were centrifuged for 10 min in 40% trichloroacetic acid (TCA) at 10,000 × g at 4°C. The obtained supernatants were used to measure lipid peroxidation (LPO) at 532 nm and expressed as nmol (g tissue)^–1^.

The remaining samples were used for measurement of antioxidant parameters. Samples were prepared in 5 volumes of 25 mmol/L sucrose containing 10 mmol/L Tris–HCl, pH 7.5 at 4°C for homogenization (Lionetto et al., 2003) using an IKA-Werk Ultra-Turrax homogenizer (Janke and Kunkel, Staufen, Germany). In the next step, samples were sonicated for 30 s at 20 kHz on ice. The sonicates were centrifuged at 5,000 × g for 10 min in 10% sulfosalicylic acid for determination of total glutathione (GSH) concentration, and at 100,000 × g for 90 min at 4°C in a Beckman ultracentrifuge for determination of the protein content and antioxidant parameters (Takada et al., 1982).

The protein content in juveniles was measured at 500 nm according to the method described by Lowry et al. (1951) with bovine serum albumin as a standard. The total activity of superoxide dismutase (SOD) was recorded as the autoxidation of adrenaline to adrenochrome at 480 nm (Misra and Fridovich, 1972). The enzymatic activity of catalase (CAT) was defined as the rate of hydrogen peroxide (H_2_O_2_) decomposition at a wavelength of 240 nm (Claiborne, 1984). The method of Tamura et al. (1982) was applied to estimate the activity of glutathione peroxidase (GSH-Px). The method is based on the oxidation of nicotinamide adenine dinucleotide phosphate (NADPH) as the substrate with tert-butyl hydroperoxide at 340 nm. The rate of NADPH oxidation and the same wavelength were used for measuring glutathione reductase (GR) activity (Glatzle et al., 1974). In this assay, the capability of GR to catalyze the recovery of reduced GSH from oxidized glutathione (GSSG) was used. The protocol described by Habig et al. (1974), which is based on the interaction of the SH group of GSH with 1-chloro-2,4-dinitrobenzene (CDNB) at 340 nm, was used to measure glutathione S-transferase (GST) activity. The enzymatic activities are given as U (mg protein) ^–1^. The total glutathione (GSH) concentration was determined at 412 nm according to the enzymatic recycling method, GSH oxidation [by 5,5′ -dithiobis-(2-nitrobenzoic acid) (DTNB)] and NADPH reduction in the presence of GR (Griffith, 1980). The concentration of sulfhydryl (SH) groups was recorded at 340 nm according to the Ellman (1959) assay. The concentration of GSH is presented as nmol (g tissue) ^–^, and of SH groups as μmol (mg tissue)^−1^.

A UV-VIS spectrophotometer (UV-1,800, Shimadzu, Japan) was used for all measurements at a measuring temperature of 25°C.

### Statistical Analyses

Homogeneity of variances, possible outliers and normality of data prior to ANOVA test application were analyzed. Factorial ANOVA was performed on oxidative stress parameters to determine the differences between two factors: the water regime (desiccation and constant high water) and the feeding regime (fasting and feeding), and their interaction. The parameter that exhibited significant interaction between factors (water regime × feeding regime) was further tested with pairwise multiple comparisons—Tukey’s test, whereas for parameters with significant differences for each factor, the *post hoc* Tukey HSD test was performed in order to determine differences among the examined groups. ANOVA was used to test the differences in biometric parameters from the start of the fasting experiment, while for data collected at the end of the experiment, ANCOVA with values of the body size parameters from the beginning as a covariate was applied. The significance level was set as *P* ≤ 0.05 for all analyses. XLSTAT, Ver. 2014.5.03 (Addinsoft, 2015) was used for pairwise multiple comparisons, and other tests were performed in STATISTICA 8.0 (StatSoft Inc, 2007).

## Results

### Biometric Parameters

**FIGURE 1:**
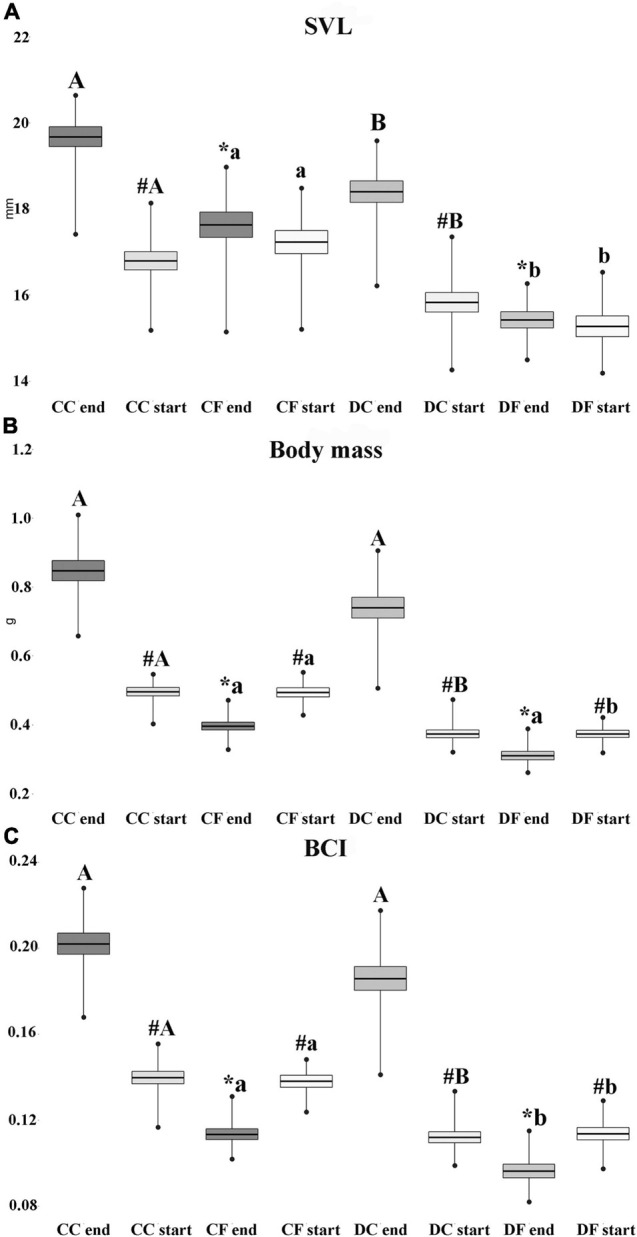
Biometric parameters: snout-vent length [**(A)**– SVL in mm], body mass [**(B)**– BM in g] and body condition index [**(C)**– BCI] at the start and end of the fasting/feeding treatment for individual that developed under conditions of desiccation and constant water. CC, feeding individuals that developed under constant water availability; CF, fasting individuals that developed under constant water availability; DC, feeding individuals that developed under exposure to the desiccation regime; DF, fasting individuals that developed under the desiccation regime. SVL and BM are given as mean ± standard error of raw data, BCI on log-transformed data. “#” indicates significant differences between same groups at the end and start of treatment; “*” indicates significant differences between fasting and feeding treatments under the same water regime; capital letters “A” and “B” indicate differences between feeding individuals exposed to desiccation and constant water availability (start vs. start, and end vs. end); lower case letters “a” and “b” indicate differences between fasting individuals exposed to desiccation and constant water (start vs. start, and end vs. end). Different letters indicate significant differences (*P* ≤ 0.05). Line, Mean value; box, Standard error; bars, Minimal and maximal value.

The duration of larval development in desiccation (44.41 ± 0.29 days) and constant water (43.92 ± 0.38 days) levels did not differ significantly (*F* = 1.04, *P* = 0.3085). Figure 1 contains data (mean ± standard error) for the biometric parameters (SVL, BM, BCI) of the examined groups. The results for biometric parameters at the beginning of the fasting/feeding treatment revealed significant differences between individuals from different developmental water regimes (constant water vs. desiccation). Individuals reared at constant high water (start CC and start CF groups) had higher values for all biometric parameters as compared to ones exposed to desiccation during development (start DC and start DF).

Results at the end of the 2-week fasting/feeding period showed that feeding juveniles that were reared under constant water (end CC) had significantly higher values for SVL in comparison to individuals that were also under feeding regimes but were exposed to desiccation (end DC) (Figure 1). No significant differences between those groups were reported for BM and BCI. Comparison among fasting groups that developed under different water regimes (end CF and end DF) revealed significant differences in SVL and BCI. For both parameters, individuals that were from constant high water displayed higher values. Juveniles that were feeding regardless of the water regime (end CC and end DC) had higher values for biometric parameters in comparison to the fasting ones (end CF and end DF) (Figure 1). The percentage of SVL growth during the 2-week treatment revealed that individuals from the different water regimes and under the feeding treatment did not differ significantly (CC = 2.085 ± 0.158%, DC = 2.315 ± 0.171%; *F* = 0.964, *P* = 0.334). Results for mass gain showed that feeding individuals pre-exposed to desiccation gained more mass in comparison to individuals reared under constant high water (CC = 71.963 ± 6.523%, DC = 99.259 ± 7.895%; *F* = 7.10, *P* = 0.0126). No significant differences in the mass loss were reported between water regimes under fasting conditions (CF = 19.636 ± 1.838%, DF = 17.086 ± 1.684%; *F* = 1.033, *P* = 0.322).

The biometric parameters from the beginning and the end of treatment showed that feeding individuals from both desiccation and constant water groups (CC and DC) increased significantly all examined parameters as compared with starting values (Figure 1). As for fasting treatment (CF and DF), individuals that exhibited food deprivation regardless of the water regime significantly decrease their body mass and BCI in comparison to values from the beginning of the treatment (Figure 1).

### Parameters of Oxidative Status

The results for factorial ANOVA analyses are provided in Table 1. Significant differences for the factors water and feeding regime as well for their interactions were observed for SOD, GSH and GST. Parameters that showed significant differences for the feeding regime factor and the interaction water x feeding regime were GSH-Px and SH groups. GR displayed statistical significance for both factors but not for interaction, while LPO was between the fasting and feeding treatments. The absence of any differences was reported only for CAT activity. Table 2 contains comparisons performed on parameters that displayed significant interaction of the water × feeding regime.

**TABLE 1:** Factorial ANOVA on examined oxidative status parameters between the water regime (desiccation and constant water availability), feeding regime (fasting and feeding), and their interaction (water regime × feeding regime).

**Parameter**	**Effect**	***F***	***P***
SOD	**Water regime**	**22.832**	**<0.0001**
	**Feeding regime**	**224.834**	**<0.0001**
	**Water regime × feeding regime**	**12.936**	**0.0009**
CAT	Water regime	1.136	0.2919
	Feeding regime	1.731	0.1947
	Water regime × feeding regime	0.329	0.5688
GSH-Px	Water regime	3.718	0.0599
	**Feeding regime**	**51.822**	**<0.0001**
	**Water regime × feeding regime**	**5.083**	**0.0289**
GSH	**Water regime**	**24.772**	**<0.0001**
	**Feeding regime**	**175.240**	**<0.0001**
	**Water regime × feeding regime**	**7.505**	**0.0087**
GR	**Water regime**	**9.750**	**0.0031**
	**Feeding regime**	**11.914**	**0.0012**
	Water regime × feeding regime	2.803	0.1007
GST	**Water regime**	**5.529**	**0.0229**
	**Feeding regime**	**93.542**	**<0.0001**
	**Water regime × feeding regime**	**4.093**	**0.0488**
SH	Water regime	2.564	0.1160
	**Feeding regime**	**62.067**	**<0.0001**
	**Water regime × feeding regime**	**19.304**	**<0.0001**
LPO	Water regime	0.266	0.6088
	**Feeding regime**	**38.341**	**<0.0001**
	Water regime × feeding regime	1.385	0.2458

*Statistical difference (P ≤ 0.05) is given in bold.*

**TABLE 2:** Multiple pairwise comparisons of oxidative stress parameters that displayed significant interaction with water regime × feeding regime (SOD, GSH-Px, GSH, GST, and SH groups).

**Contrast**	**SOD**	**GSH-Px**	**GSH**	**GST**	**SH**
Desiccation*feeding vs. constant water*feeding	**<0.0001**	**0.0021**	**<0.0001**	**0.0013**	**<0.0001**
Desiccation*feeding vs. desiccation*fasting	**<0.0001**	**0.0012**	**<0.0001**	**<0.0001**	**<0.0001**
Constant water*feeding vs. constant water*fasting	**<0.0001**	**<0.0001**	**<0.0001**	**<0.0001**	**0.016**
Desiccation*fasting vs. constant water*fasting	0.4033	0.8326	0.1515	0.8315	0.0752
Constant water*feeding vs. desiccation*fasting	**<0.0001**	**<0.0001**	**<0.0001**	**<0.0001**	**<0.0001**
Desiccation*feeding vs. constant water*fasting	**<0.0001**	**0.0004**	**<0.0001**	**<0.0001**	**<0.0001**

*All interactions that are in concordance with the set aim of this study are highlighted; significant P-values are in bold.*

**FIGURE 2:**
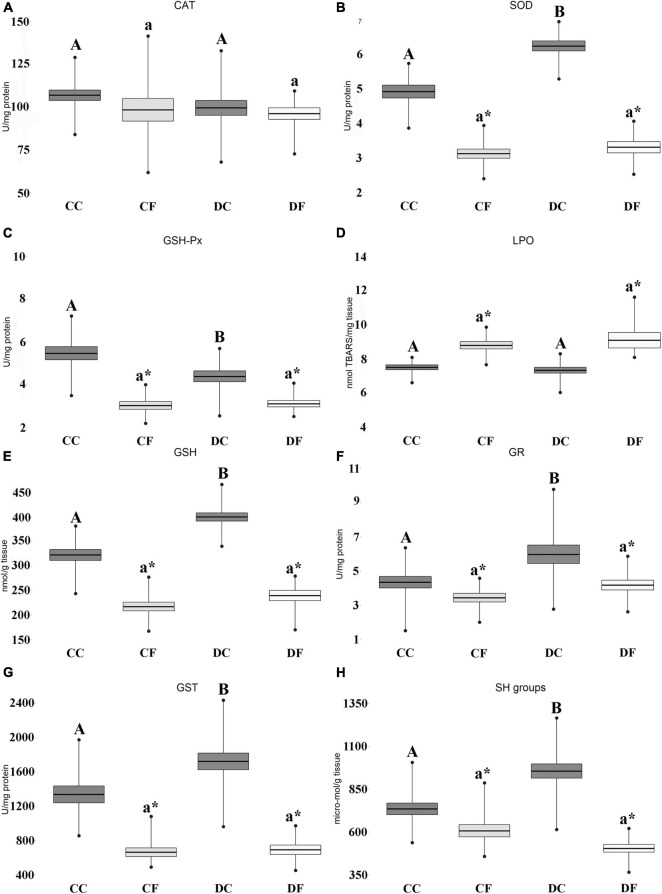
Effects of 2-weeks of fasting/feeding treatment on oxidative stress parameters of the yellow belly toad (*Bombina variegata*) juveniles that developed under constant and decreasing water levels. CC, Feeding individuals that developed under constant water availability; CF, Fasting individuals that developed under constant water availability; DC, Feeding individuals that developed under exposure to the desiccation regime; DF, Fasting individuals that developed under the desiccation regime. **(A)** Catalase; **(B)** superoxide dismutase; **(C)** glutathione peroxidase; **(D)** lipid peroxidation; **(E)** glutathione; **(F)** glutathione reductase; **(G)** glutathione-S transferase; **(H)** SH groups. “*” indicates significant differences between fasting and feeding treatments under the same water regime; capital letters “A” and “B” indicate differences between feeding individuals exposed to desiccation and constant water availability; lower case letters “a” and “b” indicate differences between fasting individuals exposed to desiccation and constant water. Different letters indicate significant differences (*P* ≤ 0.05). Line, Mean value; box, Standard error; bars, Minimal and maximal value.

Results of *post hoc* analyses conducted on parameters that showed significant differences for the examined factors or interaction are presented in Figure 2. Pre-exposure to desiccation significantly affected oxidative stress parameters only in juvenile individuals that were under feeding treatment (CC vs. DC), while no significant differences were observed for the fasting treatments (CF vs. DF) (Figure 2). Juveniles that were pre-exposed to the desiccation regimes (DC) displayed higher values of SOD, GSH, GST, GR, and SH groups as compared to the controls (CC). GSH-Px was the only parameter that was lower in pre-desiccated juveniles in comparison to individuals reared under constant water availability.

Comparison between fasting and feeding juveniles revealed that the investigated parameters changed in the same manner for both desiccation and constant water treatments (Figure 2). The 2-week-fasting juveniles (DF and CF) had significantly lower activities of SOD, GSH-Px, GR, GST and concentrations of GSH and SH groups as compared to juveniles that did not face food unavailability (DC and CC). The parameter of oxidative lipid damage (LPO) presented the opposite trend, with a significant increase in individuals exposed to fasting compared to those exposed to feeding.

## Discussion

The desiccation risk in some anuran species affects the larval period (Newman, 1989; Denver, 1997; Lind and Johansson, 2011; Richter-Boix et al., 2011; Székely et al., 2017). In most cases, escape from an unfavorable aquatic environment reduces larval periods and the quality of metamorph phenotypes (resulting in a smaller size and a lower body condition index) (Gomez-Mestre et al., 2013; Burraco et al., 2017; Kohli et al., 2019; Székely et al., 2020; Petrović et al., 2021). In *B. variegata*, pond drying leads to a significantly lower body size (SVL, BM and BCI) in comparison to individuals developing in constant water availability, but with no acceleration in developmental rate. Our results, in conjunction with previous studies conducted on the same species under desiccation-simulated scenarios, point to the presence of a trade-off in body size at metamorphosis (Sinsch et al., 2020; Petrović et al., 2021). The lower body size of juveniles is associated with a lower resistance to food absence due to low lipid reserves (Bouchard et al., 2016), postponed ossification (Arendt and Wilson, 2000), and decreased immunological function (Kohli et al., 2019). Since larval conditions predict the juvenile phenotype, we examined the carry-over effects of desiccation stress experienced by tadpoles on the oxidative status of juveniles under fasting and feeding conditions. Studying the impact of stressful conditions during development on the ability of juveniles to respond to subsequent stress is important in understanding the challenges that amphibians face in an unpredictable environment.

Under favorable feeding conditions, the oxidative status of *B. variegata* juveniles from the desiccation regime showed a significant increase in AOS components and the absence of any increased lipid oxidative damage as compared to individuals that developed under constant water availability. A buffered AOS (GSH-Px and GR) and the absence of oxidative damage in response to pond drying were also reported in the case of freshly metamorphosed *Pelobates cultripes* (Burraco et al., 2017). The observed antioxidant response of juveniles reared under decreasing water levels can be triggered by an increased metabolic rate as the consequence of compensatory growth. Even though juveniles that experienced desiccation had smaller body sizes (SVL, body mass and BCI) after metamorphosis, 2 weeks of feeding lowered these changes, especially in body mass. These individuals gained more body mass, almost doubling by the end of the experiment, indicating greater feeding activity in comparison to juveniles that were exposed to non-stressed conditions throughout the experiment. An increase in mass was expected as fat storage is crucial for survival of the upcoming hibernation process (Reading, 2007; Scott et al., 2007). Székely et al. (2020) confirmed the ability of smaller-sized individuals to compensate for their initial disadvantages. Compensatory growth could be achieved through changes in behavior, increased and longer foraging and changed digestive activity (Tarvin et al., 2015; Buhaciuc et al., 2017), or by modification of the feeding apparatus that allowed for digestion of larger prey and/or a greater amount of food (Bouchard et al., 2016). All of these activities are related to increased metabolic and mitochondrial activities, which lead to higher production of ROS (Metcalfe and Monaghan, 2001; Metcalfe and Alonso-Alvarez, 2010; Burraco et al., 2020b). However, the disturbed redox balance of desiccation experienced by juveniles and caused by a higher metabolic rate appeared to be managed by the AOS. We observed higher activity of SOD, an enzyme that protects against the superoxide anion, the primary radical produced by the mitochondria. Aside from SOD, the activation of the GSH system parameters (total GSH, GR, and GST) supported by the increased levels of thiol groups in juveniles overwhelmed the increase in ROS concentrations and prevented serious oxidative damage. The GSH system is an important part of the cell redox system that scavenges ROS directly through GSH or indirectly as a cofactor for GSH-dependent enzymes (Halliwell and Gutteridge, 2015). Compensatory strategies for delayed hatching of *Rana temporaria* larvae were also followed by activation of the antioxidant machinery and lower LPO levels, which indicate that low lipid peroxidation was favored by increased antioxidant activities (Burraco et al., 2020b).

Early life exposure to a stressor, in this case desiccation, will exert a negative or positive effect on the oxidative status during other life stages depending on the severity of the stressor and the environment during formative life (Costantini et al., 2012, 2014). Desiccation stress activates in larvae a physiological response that is mediated by the HPI axis and the AOS (Gomez-Mestre et al., 2013; Burraco et al., 2017; Petrović et al., 2021). In our previous study, the larvae of *B. variegata* under decreasing water exposure experienced higher levels of oxidative stress during metamorphic climax than individuals developing under constant water availability (Petrović et al., 2021). Individuals at Gosner’s stage 46 from the desiccation treatment group exhibited increased LPO and lower values of GSH, CAT, GR, GST and SH groups in comparison to the control. Based on the ability of desiccation to alter the oxidative status in tadpoles, the increase in AOS in juveniles that developed under desiccation could be explained as a consequence of prior exposure to stress (Oliveira et al., 2018). Costantini et al. (2014) suggested that the response to stress in early life may be beneficial for an organism’s fitness when the organism encounters the stressor again, whereas phenotypic adjustments resulting in a stress response could present a certain cost if there is no subsequent exposure to stress in adulthood. This raises the question of whether boosting the AOS in pre-desiccation juveniles provides oxidative protection against other stressors or whether it represents a cost since maintenance of the AOS is demanding energy-wise. With this in mind, yellow belly toad juveniles were subjected to food deprivation stress in order to determine how the oxidative status is modified depending on the body size and the developmental water regime. Our initial assumption was that the stress experienced during larval development would negatively affect the oxidative status of juveniles. Effects were expected due to the capability of desiccation to reduce body size and the energy reserves in juveniles, thereby limiting the energy necessary for antioxidant protection from fasting-induced stress. Fasting in anuran species is common, especially in freshly metamorphosed individuals in the wild (Geise and Linsenmair, 1988; Scott et al., 2007). At this life stage, it is caused by adaptation to the new terrestrial environment when individuals rely on their energy reserves (Nicieza et al., 2006; Bouchard et al., 2016). Fasting is associated with metabolic depression, reduced mitochondrial respiratory capacities and oxygen fluctuations, which induce increased ROS production and oxidative stress (McCue, 2010; Salin et al., 2018; Ensminger et al., 2021; Metcalfe and Olsson, 2021). Food deprivation or low intake of endogenous antioxidants is also reflected in the ability of organisms to equilibrate their oxidative status (De Block and Stoks, 2008). Cabrera-Guzmán et al. (2020) suggested a role for anuran diet composition on the antioxidant and immune responses to stressors. The functioning of some antioxidants is dependent on components that are driven directly from nutrients (GSH from cysteine precursor amino acids, GSH-Px from selenium, and SOD from copper and zinc) (Isaksson et al., 2011). Our results showed that short-term fasting in yellow belly toad juveniles induced oxidative stress when compared to fed individuals, regardless of the water regime. However, a comparison of smaller-body juveniles exposed to pre-desiccation and individuals exposed to a constant water regime but under food absence revealed no significant differences in the examined parameters, suggesting the lack of hormesis in oxidative stress response. This was opposite to our results observed on pre-desiccated juveniles (in comparison to non-desiccated), that exhibited better antioxidant response to following acute chemical stressor (pesticide deltamethrin) (Radovanović et al., 2021).

Food-deprived individuals from both the desiccation and constant water availability groups displayed a diminished antioxidant response, reduced mitochondrial antioxidant protection (SOD and GSH-Px) and second-line defense (GSH, GR, SH groups, and GST), as well as increased susceptibility to lipid oxidative damage. These results are in agreement with our study conducted on fasting larvae of crested newts (*Triturus* sp.) (Prokić et al., 2021). Two weeks of food deprivation also led to oxidative damage of lipids with a similar pattern of AOS change (SOD, GSH-Px, CAT, GST, and GSH) as observed in *B. variegata* juveniles, confirming that oxidative stress can be considered as the cost of short-term energetic benefits of reduced metabolism in response to fasting. A similar pattern of changes in oxidative status to impose energy-limited conditions regardless of body size, stress pre-exposure and in both anuran and tailed amphibian species suggests that fasting in amphibians activates a strong and specific response.

## Conclusion

In conclusion, exposure to pond drying-out during larval development reduces the body size of juveniles and induces compensatory growth under feeding conditions. In terms of oxidative stress, the carry-over effects of development under a decreasing water level could be observed as a boosted AOS in juveniles under benign conditions. Changes in water level and body size did not produce a substantial effect on the response to fasting-induced stress during the juvenile stage. This implies an ability of individuals with lower fitness to invest sufficient energy to maintain levels of oxidative stress that match those of larger-bodied individuals exposed to food deprivation. Overall, this study provides evidence that stressful conditions experienced during tadpole development can to some extent reflect on the fitness and oxidative stress of juveniles. Therefore, the hypothesis that metamorphic body size influences later life stages should be considered with caution. The questions that remain to be studied are: could the changes in the body size and oxidative stress of juveniles exposed to desiccation developmental stress seen in the laboratory have repercussions in harsher and more dynamic natural conditions; and the possible long-term or delayed adverse effects.

## Data Availability Statement

The raw data supporting the conclusions of this article will be made available by the authors, without undue reservation.

## Ethics Statement

The animal study was reviewed and approved by the Ministry of Environmental Protection of the Republic of Serbia (Permit No. 353-01-2876/2019-04), Veterinary Directorate, Department of Animal Welfare, Ministry of Agriculture, Forestry and Water Management of the Republic of Serbia (Approval No. 323-07-08393/2020-05/4).

## Author Contributions

MP, TP, and NT contributed to the conception and design of the study. MP organized the database and performed the statistical analysis. MP and TP wrote the first draft of the manuscript. MP, TP, BG, SD, JG, AK, NT, TV, and TR performed the investigation and experimental procedures. AK, TV, and NT performed the resources and experimental setup. MP and NT supervised the work. All authors contributed to writing, reviewing and editing the final version of the manuscript and approved the submitted version.

## Conflict of Interest

The authors declare that the research was conducted in the absence of any commercial or financial relationships that could be construed as a potential conflict of interest.

## Publisher’s Note

All claims expressed in this article are solely those of the authors and do not necessarily represent those of their affiliated organizations, or those of the publisher, the editors and the reviewers. Any product that may be evaluated in this article, or claim that may be made by its manufacturer, is not guaranteed or endorsed by the publisher.

## References

[B1] Addinsoft XLSTAT (2015). *Data Analysis and Statistics Software for Microsoft Excel.* Paris: Addinsoft.

[B2] Agudelo-Cantero G. A. Navas C. A. (2019). Interactive effects of experimental heating rates, ontogeny and body mass on the upper thermal limits of anuran larvae. *J. Therm. Biol.* 82 43–51. 10.1016/j.jtherbio.2019.03.010 31128658

[B3] Allen R. G. (1991). Oxygen-reactive species and antioxidant responses during development: the metabolic paradox of cellular differentiation. *Proc. Soc. Exp. Biol. Med.* 196 117–129. 1990401 10.3181/00379727-196-43171a

[B4] Álvarez D. Nicieza A. (2002). Effects of induced variation in anuran larval development on postmetamorphic energy reserves and locomotion. *Oecologia* 131 186–195. 10.1007/s00442-002-0876-x 28547685

[B5] Arendt J. D. Wilson D. S. (2000). Population differences in the onset of cranial ossification in pumpkinseed (*Lepomis gibbosus*), a potential cost of rapid growth. *Can. J. Fish. Aquat. Sci.* 57 351–356. 10.1139/f99-250

[B6] Biancardi C. M. Di Cerbo A. R. (2010). Morphometric study on tadpoles of *Bombina variegata* (Linnaeus, 1758) (Anura; Bombinatoridae). *Acta Herpetol.* 5 223–231.

[B7] Bouchard S. S. O’Leary C. J. Wargelin L. J. Charbonnier J. F. Warkentin K. M. (2016). Post-metamorphic carry-over effects of larval digestive plasticity. *Funct. Ecol.* 30 379–388. 10.1016/bs.amb.2015.07.001 26320615

[B8] Buhaciuc E. Székely P. Băncilă R. Cogălniceanu D. (2017). Food availability influences postmetamorphic growth in two spadefoot toad species (genus Pelobates). *Amphibia-Reptilia* 38 41–48.

[B9] Burraco P. Díaz-Paniagua C. Gomez-Mestre I. (2017). Different effects of accelerated development and enhanced growth on oxidative stress and telomere shortening in amphibian larvae. *Sci. Rep.* 7:7494. 10.1038/s41598-017-07201-z 28790317 PMC5548762

[B10] Burraco P. Orizaola G. Monaghan P. Metcalfe N. (2020a). Climate change and ageing in ectotherms. *Glob. Change Biol.* 26 5371–5381. 10.1111/gcb.15305 32835446

[B11] Burraco P. Valdés A. E. Orizaola G. (2020b). Metabolic costs of altered growth trajectories across life transitions in amphibians. *J. Anim. Ecol.* 89 855–866. 10.1111/1365-2656.13138 31693168

[B12] Cabrera-Guzmán E. Díaz-Paniagua C. Gomez-Mestre I. (2020). Differential effect of natural and pigment-supplemented diets on larval development and phenotype of anurans. *J. Zool.* 312 248–258. 10.1111/jzo.12827

[B13] Claiborne A. (1984). “Catalase activity,” in *Handbook of Methods for Oxygen Radical Research*, ed. Greenwald R. A. (Boca Raton, FL: CRC Press Inc), 283–284.

[B14] Costantini D. Monaghan P. Metcalfe N. B. (2012). Early life experience primes resistance to oxidative stress. *J. Exp. Biol.* 215 2820–2826. 10.1242/jeb.072231 22837454

[B15] Costantini D. Monaghan P. Metcalfe N. B. (2014). Prior hormetic priming is costly under environmental mismatch. *Biol. Lett.* 10:20131010. 10.1098/rsbl.2013.1010 24522630 PMC3949371

[B16] De Block M. Stoks R. (2008). Compensatory growth and oxidative stress in a damselfly. *Proc. R. Soc. B* 275 781–785. 10.1098/rspb.2007.1515 18182373 PMC2596904

[B17] Denver R. J. (1997). Proximate mechanisms of phenotypic plasticity in amphibian metamorphosis. *Am. Zool.* 37 172–184. 10.1093/icb/37.2.172 9154437

[B18] Eikenaar C. Isaksson C. Hegemann A. (2018). A hidden cost of migration? Innate immune function versus antioxidant defense. *Ecol. Evol.* 8 2721–2728. 10.1002/ece3.3756 29531689 PMC5838071

[B19] Ellman G. L. (1959). Tissue sulfhydryl groups. *Arch. Biochem. Biophys.* 82 70–77. 10.1016/0003-9861(59)90090-6 13650640

[B20] Ensminger D. Salvador-Pascual A. Arango B. Allen K. Vázquez-Medina J. (2021). Fasting ameliorates oxidative stress: a review of physiological strategies across life history events in wild vertebrates. *Comp. Biochem. Physiol. Part A Mol. Integr.* 256:110929. 10.1016/j.cbpa.2021.110929 33647461

[B21] Gavrić J. P. Despotović S. G. Gavrilović B. R. Radovanović T. B. Petrović T. G. Ajduković M. (2021). Oxidative stress parameters in goitrogen-exposed crested newt larvae (*Triturus* spp.): arrested metamorphosis. *Int. J. Environ. Res. Public Health* 18:9653. 10.3390/ijerph18189653 34574576 PMC8464833

[B22] Gavrilović B. R. Petrović T. G. Radovanović T. B. Despotović S. G. Gavrić J. P. Krizmanić I. I. (2021). Hepatic oxidative stress and neurotoxicity in Pelophylax kl. esculentus frogs: influence of long-term exposure to a cyanobacterial bloom. *Sci. Total Environ.* 750:141569. 10.1016/j.scitotenv.2020.141569 32853936

[B23] Geise W. Linsenmair K. E. (1988). Adaptations of the reed frog *Hyperolius viridiflavus* (Amphibia, Anura, Hyperoliidae) to its arid environment. *Oecologia* 77 327–338. 10.1007/bf00378038 28311945

[B24] Gervasi S. Foufopoulos J. (2007). Costs of plasticity: responses to desiccation decrease post-metamorphic immune function in a pond-breeding amphibian. *Funct. Ecol.* 22 100–108.

[B25] Glatzle D. Vuilleumier J. P. Weber F. Decker K. (1974). Glutathione reductase test with whole blood, a convenient procedure for the assessment of the riboflavin status in humans. *Experientia* 30 665–667. 10.1007/BF01921531 4151937

[B26] Gomez-Mestre I. Kulkarni S. Buchholz D. (2013). Mechanisms and consequences of developmental acceleration in tadpoles responding to pond drying. *PLoS One* 8:e84266. 10.1371/journal.pone.0084266 24358352 PMC3865288

[B27] Gosner K. L. (1960). A simplified table for staging anuran embryos and larvae with notes on identification. *Herpetologica* 16 183–190.

[B28] Griffith O. W. (1980). Determination of glutathione and glutathione disulfide using glutathione reductase and 2-vinylpyridine. *Anal. Biochem.* 106 207–212. 10.1016/0003-2697(80)90139-6 7416462

[B29] Guxens M. Aguilera I. Ballester F. Estarlich M. Fernández-Somoano A. Lertxundi A. (2012). Prenatal exposure to residential air pollution and infant mental development: modulation by antioxidants and detoxification factors. *Environ. Health Perspect.* 120 144–149. 10.1289/ehp.1103469 21868304 PMC3261939

[B30] Habig W. H. Pabst M. J. Jakoby W. B. (1974). Glutathione S-transferases. The first enzymatic step in mercapturic acid formation. *J. Biol. Chem.* 249 7130–7139. 4436300

[B31] Halliwell B. Gutteridge J. M. (2015). *Free Radicals in Biology and Medicine*, 4th Edn. Oxford: Oxford University Press.

[B32] Hartel T. Nemes S. Mara G. (2007). Breeding phenology and spatio-temporal dynamics of pond use by the yellow-bellied toad (*Bombina variegata*) population: the importance of pond availability and duration. *Acta Zool. Litu.* 17 56–63.

[B33] Isaksson C. Sheldon B. C. Uller T. (2011). The challenges of integrating oxidative stress into life-history biology. *Bioscience* 61 194–202.

[B34] Janssens L. Stoks R. (2018). Rapid larval development under time stress reduces adult life span through increasing oxidative damage. *Funct. Ecol.* 32 1036–1045. 10.1111/1365-2435.13068

[B35] Kohli A. Lindauer A. Brannelly L. Ohmer M. Richards-Zawacki C. Rollins-Smith L. (2019). Disease and the drying pond: examining possible links among drought, immune function, and disease development in amphibians. *Physiol. Biochem. Zool.* 92 339–348. 10.1086/703137 30990770

[B36] Labocha M. K. Schutz H. Hayes J. P. (2014). Which body condition index is best? *Oikos* 123 111–119. 10.1111/j.1600-0706.2013.00755.x

[B37] Lind M. I. Johansson F. (2011). Testing the role of phenotypic plasticity for local adaptation: growth and development in time-constrained Rana temporaria populations. *J. Evol. Biol.* 24 2696–2704. 10.1111/j.1420-9101.2011.02393.x 21954876

[B38] Lionetto M. G. Caricato R. Giordano M. E. Pascariello M. F. Marinosci L. Schettino T. (2003). Integrated use of biomarkers (acetylcholineesterase and antioxidant enzyme activities) in *Mytilus galloprovincialis* and *Mullus barbatus* in an Italian coastal marine area. *Mar. Pollut. Bull.* 46 324–330. 10.1016/S0025-326X(02)00403-4 12604066

[B39] Lowry O. H. Rosebrough N. J. Farr A. L. Randall R. J. (1951). Protein measurement with the Folin phenol reagent. *J. Biol. Chem.* 193 265–275. 14907713

[B40] McCue M. D. (2010). Starvation physiology: reviewing the different strategies animals use to survive a common challenge. *Comp. Biochem. Physiol. Part A Mol. Integr.* 156 1–18. 10.1016/j.cbpa.2010.01.002 20060056

[B41] Metcalfe N. Alonso-Alvarez C. (2010). Oxidative stress as a life-history constraint: the role of reactive oxygen species in shaping phenotypes from conception to death. *Funct. Ecol.* 24 984–996. 10.1111/j.1365-2435.2010.01750.x

[B42] Metcalfe N. Olsson M. (2021). How telomere dynamics are influenced by the balance between mitochondrial efficiency, reactive oxygen species production and DNA damage. *Mol. Ecol.* [Epub ahead of print]. 10.1111/mec.16150 34435398

[B43] Metcalfe N. B. Monaghan P. (2001). Compensation for a bad start: Grow now, pay later? *Trends Ecol. Evol.* 16 254–260. 10.1016/s0169-5347(01)02124-3 11301155

[B44] Misra H. P. Fridovich I. (1972). The role of superoxide anion in the autoxidation of epinephrine and simple assay for superoxide dismutase. *J. Biol. Chem.* 247 3170–3175. 10.1016/s0021-9258(19)45228-9 4623845

[B45] Moreira D. Welker A. Campos É. de Souza S. Hermes-Lima M. (2018). Subtropical hibernation in juvenile tegu lizards (*Salvator merianae*): insights from intestine redox dynamics. *Sci. Rep.* 8:9368. 10.1038/s41598-018-27263-x 29921981 PMC6008456

[B46] Moreira D. C. Paula D. P. Hermes-Lima M. (2021). Changes in metabolism and antioxidant systems during tropical diapause in the sunflower caterpillar *Chlosyne lacinia* (Lepidoptera: Nymphalidae). *Insect Biochem. Mol. Biol.* 134:103581. 10.1016/j.ibmb.2021.103581 33910100

[B47] Newman R. A. (1989). Developmental plasticity of Scaphiopus couchii tadpoles in an unpredictable environment. *Ecology* 70 1775–1787. 10.2307/1938111

[B48] Nicieza A. Alvarez D. Atienza E. (2006). Delayed effects of larval predation risk and food quality on anuran juvenile performance. *J. Evol. Biol.* 19 1092–1103. 10.1111/j.1420-9101.2006.01100.x 16780510

[B49] Oliveira M. F. Geihs M. A. França T. F. Moreira D. C. Hermes-Lima M. (2018). Is “preparation for oxidative stress” a case of physiological conditioning hormesis? *Front. Physiol.* 9:945. 10.3389/fphys.2018.00945 30116197 PMC6082956

[B50] Petrović T. Kijanović A. Kolarov Tomašević N. Gavrić J. Despotović S. Gavrilović B. (2021). Effects of desiccation on metamorphic climax in *Bombina variegata*: changes in levels and patterns of oxidative stress parameters. *Animals* 11:953. 10.3390/ani11040953 33805554 PMC8066544

[B51] Prokić M. Despotović S. Vučić T. Petrović T. Gavrić J. Gavrilović B. (2018). Oxidative cost of interspecific hybridization: a case study of two *Triturus* species and their hybrids. *J. Exp. Biol.* 221:jeb182055. 10.1242/jeb.182055 30127083

[B52] Prokić M. Gavrić J. Petrović T. Despotović S. Gavrilović B. Radovanović T. (2019). Oxidative stress in Pelophylax esculentus complex frogs in the wild during transition from aquatic to terrestrial life. *Comp. Biochem. Physiol. A Mol. Integr. Physiol.* 234 98–105. 10.1016/j.cbpa.2019.05.004 31082485

[B53] Prokić M. Petrović T. Despotović S. Vučić T. Gavrić J. Radovanović T. (2021). The effect of short-term fasting on the oxidative status of larvae of crested newt species and their hybrids. *Comp. Biochem. Physiol. A Mol. Integr. Physiol.* 251:110819. 10.1016/j.cbpa.2020.110819 33022409

[B54] Radovanović T. B. Gavrilović B. R. Petrović T. G. Despotović S. G. Gavrić J. P. Kijanović A. (2021). Impact of desiccation pre-exposure on deltamethrin-induced oxidative stress in Bombina variegata juveniles. *Comp. Biochem. Physiol. C Toxicol.* 250:109191. 10.1016/j.cbpc.2021.109191 34536572

[B55] Reading C. J. (2007). Linking global warming to amphibian declines through its effects on female body condition and survivorship. *Oecologia* 151 125–131. 10.1007/s00442-006-0558-1 17024381

[B56] Rehncrona S. Smith D. S. Akesson B. Westerberg E. Siesjö B. K. (1980). Peroxidative changes in brain cortical fatty acids and phospholipids, as characterized during Fe2+ and ascorbic acid stimulated lipid peroxidation *in vitro*. *J. Neurochem.* 34 1630–1638. 10.1111/j.1471-4159.1980.tb11254.x 7381489

[B57] Richter-Boix A. Llorente G. A. Montori A. (2006). Effects of phenotypic plasticity on post-metamorphic traits during pre-metamorphic stages in the anuran *Pelodytes punctatus*. *Evol. Ecol. Res.* 8 309–320.

[B58] Richter-Boix A. Tejedo M. Rezende E. L. (2011). Evolution and plasticity of anuran larval development in response to desiccation. A comparative analysis. *Ecol. Evol.* 1 15–25. 10.1002/ece3.2 22393479 PMC3287374

[B59] Ruthsatz K. Dausmann K. Paesler K. Babos P. Sabatino N. Peck M. (2020). Shifts in sensitivity of amphibian metamorphosis to endocrine disruption: the common frog (Rana temporaria) as a case study. *Conserv. Physiol.* 8:coaa100. 10.1093/conphys/coaa100 33343902 PMC7735370

[B60] Salin K. Villasevil E. Anderson G. Auer S. Selman C. Hartley R. (2018). Decreased mitochondrial metabolic requirements in fasting animals carry an oxidative cost. *Funct. Ecol.* 32 2149–2157. 10.1111/1365-2435.13125 30333678 PMC6175143

[B61] Schäfer A. M. Schaefer F. Wagner T. Sinsch U. (2018). Carabid predation on *Bombina variegata* metamorphs: size at and timing of metamorphosis matter. *Salamandra* 54 222–228.

[B62] Scott D. E. Casey E. D. Donovan M. F. Lynch T. K. (2007). Amphibian lipid levels at metamorphosis correlate to post-metamorphic terrestrial survival. *Oecologia* 153 521–532. 10.1007/s00442-007-0755-6 17530291

[B63] Simon M. N. Ribeiro P. L. Navas C. A. (2015). Upper thermal tolerance plasticity in tropical amphibian species from contrasting habitats: implications for warming impact prediction. *J. Therm. Biol.* 48 36–44. 10.1016/j.jtherbio.2014.12.008 25660628

[B64] Sinsch U. Leus F. Sonntag M. Hantzschmann A. M. (2020). Carry-over effects of the larval environment on the post-metamorphic performance of Bombina variegata (Amphibia, Anura). *Herpetol. J.* 30 126–134. 10.33256/hj30.3.126134

[B65] StatSoft Inc (2007). *STATISTICA (Data Analysis Software System), version 8.0.* Available online at: www.statsoft.com (accessed May 21, 2021).

[B66] Székely D. Cogălniceanu D. Székely P. Armijos-Ojeda D. Espinosa-Mogrovejo V. Denoël M. (2020). How to recover from a bad start: size at metamorphosis affects growth and survival in a tropical amphibian. *BMC Ecol.* 20:24. 10.1186/s12898-020-00291-w 32316956 PMC7175581

[B67] Székely D. Denoël M. Székely P. Cogălniceanu D. (2017). Pond drying cues and their effects on growth and metamorphosis in a fast developing amphibian. *J. Zool.* 303 129–135.

[B68] Takada Y. Noguchit T. Kayiyama M. (1982). Superoxide dismutase in various tissues from rabbits bearing the Vx-2 carcinoma in the maxillary sinus. *Cancer Res.* 42 4233–4235. 7105016

[B69] Tamura M. Oshino N. Chance B. (1982). Some characteristics of hydrogen- and alkylhydroperoxides metabolizing systems in cardiac tissue. *J. Biochem.* 92 1019–1031. 10.1093/oxfordjournals.jbchem.a134017 6294065

[B70] Tarvin R. D. Silva Bermúdez C. Briggs V. S. Warkentin K. M. (2015). Carry-over effects of size at metamorphosis in red-eyed treefrogs: higher survival but slower growth of larger metamorphs. *Biotropica* 47 218–226.

[B71] Underwood W. Anthony R. Gwaltney-Brant S. Poison A. S. P. C. A. Meyer R. (2013). *AVMA Guidelines for the Euthanasia of Animals*, 2013 Edn. Schaumburg, IL: American Veterinary Medical Association.

